# Association between Urine Specific Gravity as a Measure of Hydration Status and Risk of Type 2 Diabetes: The Kailuan Prospective Cohort Study

**DOI:** 10.3390/nu16111643

**Published:** 2024-05-27

**Authors:** Yinqiao Dong, Shuohua Chen, Yaohui Yu, Wenjuan Li, Zhongqing Xu, Juan Du, Shan Huang, Shouling Wu, Yong Cai

**Affiliations:** 1 School of Public Health, Shanghai Jiao Tong University School of Medicine, Shanghai 200025, China; dyq1997@sjtu.edu.cn; 2 Public Health Department, Hongqiao International Institute of Medicine, Tongren Hospital, Shanghai Jiao Tong University School of Medicine, Shanghai 200335, China; 3 Department of Cardiology, Kailuan General Hospital, North China University of Science and Technology, Tangshan 063000, China; csh01062011@163.com; 4 School of Public Health, North China University of Science and Technology, Tangshan 063210, China; yuyaohui1114@163.com; 5 School of Clinical Medicine, North China University of Science and Technology, Tangshan 063210, China; 18526296082@163.com; 6 Department of General Practice, Tongren Hospital, Shanghai Jiao Tong University School of Medicine, Shanghai 200335, China; zhongqing_xu@126.com; 7 Endocrinology Department, Tongren Hospital, Shanghai Jiao Tong University School of Medicine, Shanghai 200335, China; dj1135443@163.com

**Keywords:** hydration status, type 2 diabetes, cohort study, dehydration, time-dependent

## Abstract

Diabetes, especially type 2 diabetes (T2D), poses an unprecedented challenge to global public health. Hydration status also plays a fundamental role in human health, especially in people with T2D, which is often overlooked. This study aimed to explore the longitudinal associations between hydration status and the risk of T2D among the Chinese population. This study used data from the large community-based Kailuan cohort, which included adults who attended physical examinations from 2006 to 2007 and were followed until 2020. A total of 71,526 participants who eventually met the standards were divided into five hydration-status groups based on their levels of urine specific gravity (USG). Multivariable and time-dependent Cox proportional hazards models were employed to evaluate the associations of baseline and time-dependent hydration status with T2D incidence. Restricted cubic splines (RCS) analysis was used to examine the dose–response relationship between hydration status and the risk of T2D. Over a median 12.22-year follow-up time, 11,804 of the participants developed T2D. Compared with the optimal hydration-status group, participants with dehydration and severe dehydration had a significantly increased risk of diabetes, with adjusted hazard ratios (95% CI) of 1.30 (1.04–1.63) and 1.38 (1.10–1.74). Time-dependent analyses further confirmed the adverse effects of impending dehydration, dehydration, and severe dehydration on T2D incidence by 16%, 26%, and 33% compared with the reference group. Inadequate hydration is significantly associated with increased risks of T2D among Chinese adults. Our findings provided new epidemiological evidence and highlighted the potential role of adequate hydration status in the early prevention of T2D development.

## 1. Introduction

Type 2 diabetes (T2D) contributes to significant increases in the disease burden worldwide, particularly in low- and middle-income countries (LMICs), according to GBD 2019 data [1,2]. By 2045, the Western Pacific is expected to be home to 260 million adults with diabetes—the highest diabetic population in the world [3]. In China, T2D is also an important public health problem; in 2018, the prevalence of diabetes increased to 12.4% among Chinese adults, according to a recent national survey report [4]. T2D increases the risk of cardiovascular disease (CVD) [5], cancer burden [6], and mortality from chronic diseases [7]. T2D has different etiologies, in which modifiable factors such as nutritional status and water intake play a fundamental role in the prevention of adult T2D [8,9,10,11]. However, epidemiological evidence of the relationship between T2D and individual hydration status among adults, especially in the Western Pacific region, appears limited.

Proper hydration is considered a key aspect of optimal physiological function and health of the physical body, yet it is often overlooked by many people [11]. Water plays a crucial role in human cells, and adequate hydration helps to perform carrier functions such as intake of various nutrients, functional regulation, energy production, and maintaining body vigor [12]. Currently, a systematic review summarized the long-term, adverse effects of underhydration on health outcomes, emphasizing the importance of optimizing hydration status for chronic disease prevention [13]. Moreover, some population-based studies also demonstrated that dehydration had adverse effects on chronic diseases [14,15], renal impairment [16], cognitive performance [17], cardiovascular health [18], and both all-cause and cardiovascular mortality [19]. In addition, gender- and age-specific differences and lifestyle factors in hydration status have been described in the literature and in the water-intake recommendations proposed by several national and public organizations [20,21]. Therefore, further understanding of the relationship between hydration status and human health and the identification of vulnerable populations will be important for public health and precise prevention.

Proper hydration is important to prevent the development of diabetes, as it is essential in blood glucose regulation [22]. Current biological evidence suggests that inadequate hydration status increases vasopressin (AVP) levels, thereby mediating organs such as the pancreatic islets, liver, and hypothalamus and causing imbalances in blood glucose levels, which affects the metabolic system and ultimately leads to diabetes [23]. With hydration status gaining attention, several observational studies have found significant associations between markers of dehydration and increased risk of diabetes [13]. For example, there are several studies showing associations between increased copeptin level (a surrogate measure of AVP, and therefore a measure of hydration status) and the risk of diabetes [24,25,26,27]. However, due to its complexity and high cost, the copeptin test is not normally performed in general clinical practice and large-scale population-based studies. Urine specific gravity is an easy, rapidly accessible, and inexpensive routine test used to determine hydration status in larger sample studies [28]. Therefore, it may be a useful marker of dehydration and is widely utilized in practice to identify individuals at high risk of health outcomes. Up to now, the potential predictive value of hydration status (especially measured by USG) for T2D among Asian populations has been inadequately explored in the currently available evidence. Notably, the hydration status in the body is subject to time-varying exposure, and its time-dependent effect on human health is the weighted average of the short-term effects at each time-updated interval during the follow-up [29,30]. Previous cohort studies have generally examined the long-term effects of baseline hydration status on human health. However, few studies have investigated the time-dependent effects of hydration status on human health, especially with regard to T2D. Therefore, the relationship between baseline and time-dependent hydration status and T2D incidence remains unclear.

Therefore, the objective of the current study is to prospectively investigate whether hydration status is associated with diabetes in Chinese populations. In the present study, we propose the hypothesis that inadequate hydration promotes the development of T2D. To test this hypothesis and fill this gap in the field, we employed urine specific gravity (USG), a validated biomarker of human hydration status, to investigate associations between baseline and time-dependent hydration status and T2D incidence based on an ongoing population-based, large-scale prospective cohort study with up to 15 years of follow-up.

## 2. Materials and Methods

### 2.1. Study Population

The Kailuan Study was an ongoing, prospective cohort study conducted in Tangshan, China. Briefly, this large community-based cohort study was performed on average every 2 years from June 2006 to December 2020 at 11 hospitals, and involved a total of 7 health surveys. The study design details have been documented in earlier-published articles [31,32]. Among 101,510 participants who took part in the initial survey wave in 2006–2007, we excluded individuals who had incomplete information on urine specific gravity (USG) (*n* = 22,082) and abnormal or extreme data (USG < 1.000 or USG > 1.040) (*n* = 22,082) and those suffering from cancer or chronic kidney diseases (*n* = 537). Participants with BMI > 40 at baseline were excluded because it is known that this modifies the distribution of body fluids and increases USG levels (*n* = 29). Participants with existing diabetes at baseline (*n* = 7336) were further excluded for the survival analysis. Following the exclusions, 71,526 participants were ultimately enrolled in the prospective analysis (Figure S1). 

The present study was approved by the Kailuan General Hospital Ethics Committee, China (Ethics number: 2006–05, Trial registration number: ChiCTR–TNC–11001489). The study was conducted strictly within the guidelines of the Declaration of Helsinki, and all subjects were informed and signed informed consent before every survey circle. The study data in this study were anonymous and de-identified. The study provided participants with a free general physical examination.

### 2.2. Assessment of Hydration Status

In population-based studies, USG is an alternative indicator that is used to measure the hydration status of participants, and it is widely used in clinical practice because it offers advantages such as easy, quick, and cheap testing. All participants were asked to keep fasting and not to drink water before the urine samples were collected. Morning urine samples were randomly collected in the midstream of spot urine from each participant during the 2006–2007 baseline physical examination and following seven-cycle follow-ups. USG was determined by laboratory physicians using a dry chemistry test method (H12-MA test strips, Changchun Dirui Medical Technology Co., Ltd., Changchun, China) within 2 h after urine sample collection. The central laboratory of Kailuan General Hospital used an automatic urine sediment analyzer (N-600, Dirui, Changchun, China) to analyze all urine samples. 

Participants were categorized into five hydration status subgroups based on baseline USG levels. Based on previous clinical and epidemiologic evidence, optimal hydration status was defined as USG levels less than 1.010 (reference group) [33]. Our study considered that a level greater than or equal to 1.010 indicated different degrees of hypohydration (defined as having highly concentrated urine), which were further categorized into the following groups: marginally adequate hydration (1.010 ≤ USG < 1.015, Group 2) and impending dehydration (1.015 ≤ USG < 1.020, Group 3). Based on previous literature, we further categorized the inadequate hydration status into dehydration and severely dehydration groups. Dehydration status (1.020 ≤ USG < 1.030, Group 4) was defined based on the commonly used USG threshold values [33,34,35]. Meanwhile, considering the adverse effects of dehydration in clinical practice, we used the most stringent dehydration threshold among the commonly used USG thresholds (USG ≥1.030, Group 5) to represent severe dehydration status [36]. Time-dependent USG was defined as USG levels updated by follow-up examinations (2006–2020), respectively. 

### 2.3. Definition of T2D and Follow-Up 

The main outcome—type 2 diabetes (T2D), based on ICD-10 code (E11)—was determined according to the following criteria: fasting blood glucose (FBG) ≥7.0 mmol/L, self-report of a physician diagnosis, or self-reported uses of antidiabetic medication [37]. 

The number of cases based on the 3 different methods or overlapping combinations for determining T2D is displayed as a proportional Venn diagram (Figure S2). The follow-up time was calculated from baseline to T2D onset date (first available follow-up meeting diagnostic criteria), or death date or last available survey time (31 December 2020), whichever was earlier. All incident data were obtained from Kailuan General Hospital’s Medicare system and updated at annual follow-up visits to calculate incidence rates (per 1000 person years).

### 2.4. Covariates

Sociodemographic information (e.g., gender, age, and education), lifestyle (e.g., smoking status, alcohol drinking, physical activity, and salt intake), as well as information about medication use were obtained from standard questionnaires used to interview participants before the physical examination, as detailed elsewhere [38]. Smoking status and alcohol consumption were divided into two categories based on current status (yes/no). Combined with occupational and discretionary activities, physical activity was divided into two groups with and without physical activity. Salt intake was determined from responses to questions related to salt preferences: low, medium, and high. However, it is difficult to accurately obtain salt intake (g/day) of participants in large-scale population-based studies. Therefore, we defined salt-intake subgroups based on estimated salt intake corresponding to 24 h urinary sodium excretion: low (<6 g/day), medium (6–10 g/day), and high (>10 g/day), as described previously [38,39,40]. Anthropometrics, including height, weight, and blood pressure, were measured by trained physicians. BMI was calculated as weight (kg) divided by height squared (m^2^). Detailed blood pressure measurements were described in the available studies [41]. Hypertension was defined as systolic blood pressure (SBP) ≥140 mmHg or diastolic blood pressure (DBP) ≥90 mmHg, self-reported use of anti-hypertensive medications, or self-reported history of hypertension. Laboratory tests included fasting blood glucose (FBG), hematocrit, total cholesterol (TC), triglyceride (TG), low-density lipoprotein cholesterol (LDL-C), high density lipoprotein cholesterol (HDL-C), as well as levels of creatinine, blood urea nitrogen (BUN), plasma high-sensitivity C-reactive protein (hsCRP) and other biochemical measurements. Detailed information on the collection, measurement, and analysis of blood samples can be found in previous studies [41,42]. All biochemical variables were determined in Kailuan General Hospital’s central laboratory by employing an autoanalyzer (Hitachi 747; Hitachi, Tokyo, Japan) according to standard operating methods.

### 2.5. Statistical Analysis

Baseline characteristics for participants with various degrees of hydration status, as defined by USG levels, were displayed as frequency and percentage (%), $\bar{X}$ ± SD (means and standard deviations), median (interquartile ranges, IQR), as available based on data type and distribution. To compare the baseline characteristics across different hydration status strata, the χ^2^ test was performed for categorical variables, and the parametric test (ANOVA methods) or non-parametric test (Kruskal–Wallis test) was performed for continuous variables with normal or skewed distribution, respectively. Due to skewed distribution, hsCRP and TG were log-transformed as continuous variables in the following regression models.

Multivariable Cox proportional hazards regression models were performed to calculate HRs (95% confidence intervals, CIs) to quantify the prospective associations of baseline USG with the T2D incidence, respectively. Based on previous literature using stepwise regression analysis methods, this study used three models comprehensively adjusted for potential confounders [41,43,44]. The crude model is the unadjusted model (Model 1). Model 2 was further adjusted for age (<65 and ≥65 years), sex, education, salt intake, physical activity, smoking alcohol consumption, and BMI based on Model 1. Model 3 was additionally adjusted for history of hypertension, TC, TG, hsCRP, eGFR, BUN, plasma creatinine (Cre), serum uric acid (SUA), and hematocrit based on Model 2. Considering the instability of single time-point exposure measurements at baseline, time-dependent Cox proportional-hazards regression was used to analyze the longitudinal associations of time-dependent hydration status with the T2D risk at multiple intervals [45]. The timeline of physical examinations was 2006–2020, and the follow-up time for each participant was divided into different intervals for the time-dependent analysis. In the time-dependent analysis, the covariates in the multivariate model described above (except gender and education) are considered as time-varying variables. Multiple imputation was used to impute the missing covariate data. The cumulative incidence of endpoint events was displayed graphically with Kaplan–Meier curves, and the differences across subgroups of hydration status stratified by USG were compared with log-rank tests. The proportional hazards assumptions were assessed by the Schoenfeld residual method and no potential violations were identified. 

In addition, restricted cubic spline (RCS) models were fitted to evaluate the dose–response relationship of the continuous USG index with the risk of T2D. Subgroup analyses were further stratified by gender, age (<65 and ≥65 years), alcohol consumption, and smoking to examine the possible modifying effects of different subgroups. Several sensitivity analyses were adopted to verify the consistency and robustness of the study results. First, participants who presented with study endpoints during the 2008–2009 follow-up were excluded, which addressed potential bias caused by reverse causality. Second, we further excluded underweight participants (with a BMI less than 18). Third, considering the effect of cut-off values, the present study reanalyzed relationships between hydration status and diabetes with an alternative cut-off value of severe dehydration (USG ≥ 1.025 g/mL) [46] and a new subgroup (which combined severely dehydrated and dehydrated groups). Finally, considering the clinical practice implications, this study further evaluated the effect of clinical dehydration status (i.e., severe dehydration) on T2D compared to non-clinical dehydration status (Group 1–4). All analyses were performed in the SAS 9.4 (SAS Institute Inc., Cary, NC, USA). A two-sided *p* value less than 0.05 indicated statistical significance.

## 3. Results

### 3.1. Baseline Characteristics of the Study Population

Baseline characteristics and laboratory parameters based on baseline USG index groups during the first cycle of surveys are shown in Table 1. A total of 71,526 participants were eventually included in this study, with a baseline mean age of 51.8 years. 

Compared to participants with normal USG levels, the dehydration (USG group 4) and severely dehydrated (USG group 5) groups had higher prevalences of hypertension, blood pressure, BMI, FBG, BUN, hematocrit, and lower concentrations of HDL-C and eGFR. In addition, the inadequate hydration-status subgroups were more prone to be younger, drink alcohol, smoke, consume more salt in their diet, and less likely to participate in physical activity. 

### 3.2. Prospective Association between Hydration Status and T2D

**Figure 1:**
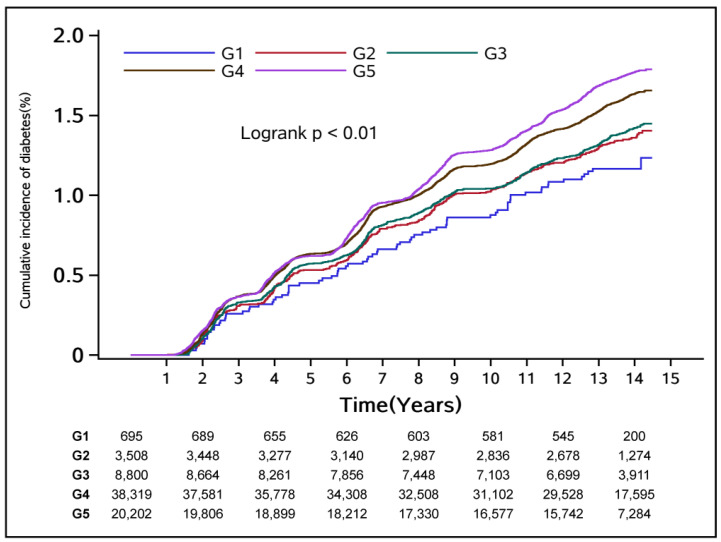
Kaplan–Meier curves of cumulative incidence of T2D among the overall population. Note: hydration status categories were based on urine specific gravity (USG) as Group 1 (G1): 1.000 ≤ USG ≤ 1.010 g/mL; Group 2 (G2): 1.010 < USG < 1.015 g/mL; Group 3 (G3): 1.015 ≤ USG < 1.020 g/mL; Group 4 (G4): 1.020 ≤ USG < 1.030 g/mL; Group 5 (G5): USG ≥ 1.030 g/mL. G1 was used as the reference group. *p* < 0.001 for differences among curves using the log-rank test.

During the median 12.22 years follow-up time, 11,084 (15.50%) of the 71,526 participants developed T2D. The Kaplan–Meier curves displayed significant differences in the cumulative incidence of T2D across different USG subgroups (log-rank test *p* < 0.001, Figure 1). The multivariate Cox regression analysis found that, with USG group 1 (G1) as a reference, the adjusted HRs (95% CI) were 1.18 (0.93–1.50), 1.20 (0.95–1.50), 1.34 (1.07–1.67), and 1.37 (1.10–1.72) for subjects with USG levels in G2–G5, respectively, in the model 2 (*p* for trend < 0.001) (Table 2). Similarly, USG group 4 (HR:1.30, 95% CI: 1.04–1.63) and USG group 5 (HR:1.38, 95% CI: 1.10–1.74) also significantly increased the risk of developing T2D in the full-adjusted model. The full results of relationships between baseline USG and T2D using multivariate Cox regression models based on the full-adjusted model are presented in Table S1.

Similarly, during the time-dependent Cox analysis, we found that time-varying hydration status was still associated with T2D incidence (Table 2). Compared with the reference group (USG group 1), impending dehydration (USG group 3), dehydration status (USG group 4), and severe dehydration (USG group 5) all increased the risk of T2D in the crude model, with HR (95% CI) of 1.19 (1.08–1.31), 1.32 (1.20–1.45), 1.41 (1.28–1.55), respectively. After adjusting for all covariates, the time-dependent effects of the different dehydration states mentioned above on T2D were also significant, increasing T2D incidence by 16%, 26%, and 33%, respectively. The full results of the fully adjusted Cox regression model were shown in Table S2.

**Figure 2:**
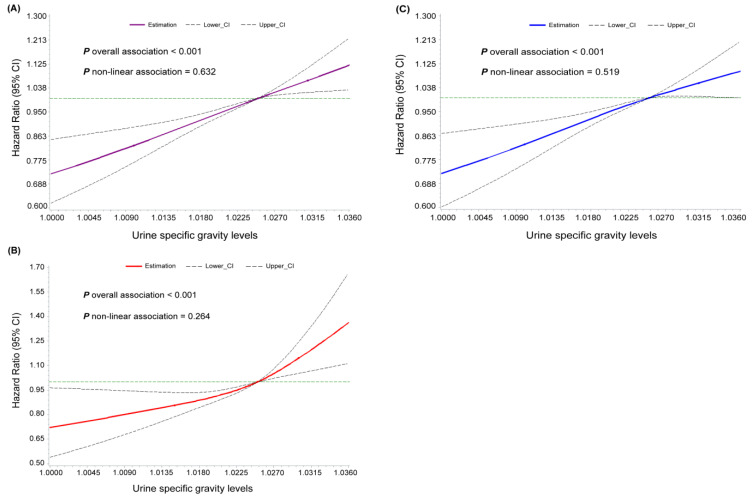
Restricted cubic spline analyses of the associations of continuous urinary specific gravity (USG) with risk of type 2 diabetes in (**A**) the overall population (**B**) female (**C**) male. Note: point estimates (solid line) and 95% confidence intervals (dashed lines) were based on Cox regression models of the restricted cubic spline with 3 knots at 10th, 50th, and 90th percentiles. All models were adjusted for sex, age, education, smoking status, alcohol drinking, physical activity, salt intake, history of hypertension, body mass index, high-sensitivity C-reactive protein, total cholesterol, triglyceride, eGFR, hemoglobin, serum uric acid, blood urea nitrogen, and plasma creatinine.

In addition, the concentration–response relationship between hydration status and the risk of developing T2D was further evaluated when USG was analyzed as a continuous variable (Figure 2). The RCS analysis revealed linear positive relationships of the risk of incident T2D with the USG index after adjusting for confounding factors among the overall population (*P* _non-linear association_ = 0.63). The concentration–response relationship between USG levels and T2D incidence in females and males with the same non-significant violation for linearity as in the overall population, respectively (*P* _non-linear association_ = 0.26 and *P* _non-linear association_ = 0.52, respectively).

### 3.3. Subgroup Analysis

**Figure 3:**
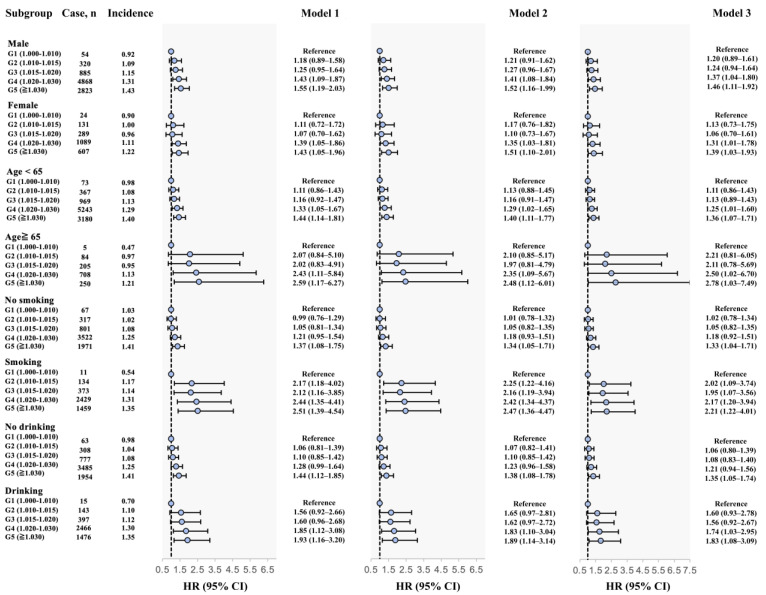
Subgroup analysis of the association between hydration status and risk of T2D based on the multivariable Cox regression models. Note: incidence rate, per 1000 person years. Model 1: crude model; Model 2: adjusted for age, gender, BMI (categorical), education, smoking, drinking, physical activity, and intake of salt based on model 1; Model 3: further adjusted for history of hypertension, total cholesterol (TC), triglyceride (TG), C-reactive protein (CRP), serum uric acid (SUA), estimated glomerular filtration rate (eGFR), blood urea nitrogen (BUN), plasma creatinine (Cre), and hematocrit based on model 2.

The relationship between the USG index and T2D incidence was stratified by gender, age, smoking, and drinking status, and the results of the subgroup analysis were summarized in Figure 3 and Table S3. Overall, USG group 4 and group 5 were also significantly associated with increased T2D risk among various subgroups, which is consistent with the main findings in the total population. Our findings further revealed that the associations of inadequate hydration status (USG group 4 and group 5) of the risk of T2D were more prominent in men and older adults (older than 65 years), as shown by the interaction effects of hydration status with sex and age (*P* _interaction_ < 0.001 in both subgroups).

### 3.4. Sensitivity Analyses

Sensitivity analyses after excluding participants who presented T2D cases within the first follow-up and those who were severely malnourished found similarly robust associations of inadequate hydration status (USG group 4 and group 5) with T2D incidence (Table S4). Consistently, these significant relationships remained even when an additional cutoff for severe dehydration (USG ≥ 1.025 g/mL) was used in the sensitivity analysis (Table S5). Similarly, the results of sensitivity analyses adjusting USG subgroups found that the highest USG group increased the risk of developing T2D (Table S6). Finally, we found that severe dehydration (USG ≥ 1.030) still increased the risk of T2D by 11% compared with non-clinical dehydration status, which was consistent with our main findings (Table S7). Therefore, the findings from several sensitivity analyses were in line with the main analysis, enhancing the robustness of our findings.

## 4. Discussion

To the best of our knowledge, the current study is the first prospective study to investigate the relationship between various hydration statuses and risk of T2D in Chinese adults in a large population-based cohort with long-term follow-up. Our findings confirmed that inadequate hydration status (dehydration and severe dehydration) was significantly associated with the risk of developing T2D. Additionally, significant linear dose–response associations between hydration status (USG as a continuous hydration index) and T2D incidence further indicated that inadequate hydration status increased the risk of T2D. Furthermore, men, older adults, smokers, and alcohol drinkers are more susceptible to increased diabetes risk due to poor hydration status. This large prospective study included more than 70,000 individuals and determined that monitoring hydration status (urine specific gravity index) may confer important value in the primary prevention of T2D and promise to be a simple and effective tool in regular clinical practice for diabetes (Figure S3).

It is widely recognized that proper hydration plays a crucial role in human health. However, the current epidemiologic evidence assessing the long-term effects of hydration status on diabetes was limited and mainly focused on European and American populations, and there is no population-based evidence from other countries. Indeed, several prospective or observational studies have reported significant associations between elevated plasma copeptin and increased future risk of diabetes [24,25,26,27]. The above studies almost found that the risk of diabetes increased with increasing quartiles of plasma copeptin compared to good hydration status (reference group, plasma copeptin < 3 pmol/L), which is consistent with our conclusions. In addition, a cross-sectional study from NHANES similarly supported our finding that a higher USG, a urinary marker of hydration status, was associated with greater risks of diabetes in U.S. adults [35]. However, no prospective association between hydration status and diabetes has been explored based on urine specific gravity. Our findings provide additional prospective associations that, compared to the reference group (USG < 1.010, corresponding to other markers of good hydration, e.g., plasma copeptin < 3pmol/L, plasma osmolality 285 mOsmol/kg, and blood sodium ions 140 mmol/L) [13], under-hydration (1.020 ≤ USG < 1.030 or 1.020 ≤ USG < 1.025) and clinical dehydration (USG ≥ 1.030 or USG ≥ 1.025) both increased the risk of T2D. Moreover, our findings emphasized that increased risk starts at levels of under-hydration that are much milder than clinical dehydration. Our findings fill the knowledge gap in the prospective association of urine specific gravity as a measure of hydration status with T2D and further provide valuable population-based evidence from China with up to 15 years of follow-up.

Moreover, findings from studies on water intake related to body hydration status and risk of diabetes can provide some indirect evidence. The previous study conducted in France over 9 years of follow-up time reported that lower daily water intake increased new-onset hyperglycemia risk, and the same trend was found in new-onset diabetes, although the relationship was not significant [47]. This finding, in turn, indicated that adequate hydration played an important role in T2D prevention. Similarly, a cross-sectional study conducted in the UK also demonstrated that higher plain water intake was associated with lower type 2 diabetes risk [48]. Therefore, water intake and excretion determine the hydration status of the body and emphasize that appropriate hydration status is crucial in preventing T2D occurrence and development. The present study further identified that dehydration induced a linear association with increased risk of T2D when hydration status assessed by USG exceeded clinical cut-off values, which is in agreement with the existing evidence.

Currently, several potential biological mechanisms can be used to explain the adverse effects of underhydration in the pathogenesis of T2D. Vasopressin, as a major regulator of water homeostasis within the body, also plays an important role in glucose homeostasis. Inadequate water intake induces elevated levels of vasopressin, which stimulates vasopressin V1b receptors on the pancreatic islets, thereby causing increased levels of glucagon and adrenocorticotropic hormone, and thus ultimately leading to diabetes [23,49]. Moreover, current evidence from the population and experiment indicated that high plasma copeptin concentrations, a surrogate marker of vasopressin, were associated with increased risk of type 2 diabetes, which also suggested another potential link between poorer hydration status and diabetes [24,50]. On the contrary, higher water intake was associated with reduced fasting glucose concentrations, copeptin concentrations, and T2D risk [51,52]. More related population-based epidemiological studies and biological mechanism studies are needed in the future to determine the underlying associations between dehydration and T2D risk.

In addition, our study found significant interactions of sex and age in the relationship between hydration status and T2D risk, revealing that men and older adults are more likely to develop T2D due to dehydration. Older adults have a greater risk of water-loss dehydration, since they are often unlikely to reach recommended water intake standards. In addition, they also have reduced body water reserves due to decreased physical performance, decreased kidney function, delayed response to thirst signals, and medications with diuretic effects [53,54]. As a result, the effect of poorer hydration status on the risk of T2D could be greater in older adults. However, while there is limited and controversial evidence for gender differences in hydration, a recent study found that there seemed to be gender differences in body water regulation, with women having higher body water levels than men [21]. In addition, the available evidence supports the idea that women have better hydration status owing to better water intake patterns in females compared to males [55], which may contribute to gender differences in hydration status. Therefore, our findings emphasize that we should not overlook the role of hydration in these susceptible populations, which can help provide targeted coping strategies for T2D primary prevention.

There are various strengths in the current research. Given the current limited evidence of hydration status and T2D, especially in the Asia–Pacific population, this study is the first study to prospectively explore the role of hydration status in T2D incidence among Chinese adults. Moreover, this study is based on a community-based cohort with a large study population and involved 15 years of follow-up to ensure that stable and reliable longitudinal associations would be reflected, as well as to fill the research gap in the Chinese population. Our study also had some limitations. Its limitations were mainly attributed to sources of selection and information bias. In terms of information bias, first, since there are many commonly used clinical indicators that can be implemented to evaluate hydration status, this study may introduce exposure misclassification bias by employing single urine specific gravity as a hydration status assessment indicator. Nevertheless, urine indicator measurements provide a convenient and practical method for evaluating hydration status in large-scale population-based studies because obtaining such measurements does not require high technical skills and urine sample collection is noninvasive, cheap, and quick to measure [28]. Second, there may be undetected factors that may affect urine specific gravity measurements and the absence of disease-specific death data, which could potentially bias the results of the study. Third, given that the Kailuan cohort data did not measure other diabetes diagnostic indicators, such as hemoglobin A1c (HbA1c), glucose tolerance tests, our findings may be affected by outcome misclassification bias, which might lead to an underestimated incidence of T2D. In addition, the definition of T2D in this study included information such as self-reported disease and medication history, so the findings may be subject to recall bias. Sensitivity analysis results using only fasting glucose to define diabetes will help reduce recall bias. Finally, the results should be extrapolated with caution, and the findings from this study have yet to be validated in other populations and cohorts. 

## 5. Conclusions

In conclusion, our study provided additional epidemiological evidence that hydration status, particularly inadequate hydration status, is associated with increased type 2 diabetes risk among Chinese adults. Findings from our research emphasized the potential role of proper hydration status in preventing the development of T2D, particularly for vulnerable populations such as men and older adults, which also provided intervention strategies and guidelines for the prevention of T2D.

## Figures and Tables

**Table 1:** Baseline characteristics of the study participants in Northern China from 2006 to 2007 (*n* = 71,526).

Characteristics	Total Population	Categories of Hydration Status Index (Urine Specific Gravity, USG)					*p* Value
		Group 1	Group 2	Group 3	Group 4	Group 5	
		1.000 ≤ USG < 1.010	1.010 ≤ USG < 1.015	1.015 ≤ USG < 1.020	1.020 ≤ USG < 1.030	USG ≥ 1.030	
	(*n* = 71,526)	(*n* = 695)	(*n* = 3508)	(*n* = 8801)	(*n* = 38,320)	(*n* = 20,202)	
Age, mean (SD), years	51.8 ± 12.6	52.0 ± 13.7	54.7 ± 13.9	54.9 ± 13.1	52.1 ± 12.3	49.2 ± 12.1	<0.001
Sex, N (%)							<0.001
Female	15,090 (21.1)	207 (29.8)	1032 (29.4)	2332 (26.5)	7603 (19.8)	3916 (19.4)	
Male	56,436 (78.9)	488 (70.2)	2476 (70.6)	6469 (73.5)	30,717 (80.2)	16,286 (80.6)	
Education, N (%)							<0.001
Below high school	57,197 (80.0)	540 (77.7)	2729 (77.8)	7024 (79.8)	31,240 (81.5)	15,664 (77.5)	
High school and above	14,329 (20.0)	155 (22.3)	779 (22.2)	1777 (20.2)	7080 (18.5)	4538 (22.5)	
Smoking, N (%)							<0.001
No	43,493 (60.8)	531 (76.4)	2546 (72.6)	6064 (68.9)	23,012 (60.1)	11,340 (56.1)	
Yes	28,033 (39.2)	164 (23.6)	962 (27.4)	2737 (31.1)	15,308 (39.9)	8862 (43.9)	
Drinking, N (%)							<0.001
No	42,881 (60.0)	522 (75.1)	2432 (69 3)	5874 (66.7)	22,751 (59.4)	11,302 (55.9)	
Yes	28,645 (40.0)	173 (24.9)	1076 (30.7)	2927 (33.3)	15,569 (40.6)	8900 (44.1)	
Physical activity, N (%)							<0.001
No	5497 (7.7)	20 (2.9)	190 (5.4)	541 (6.1)	3253 (8.5)	1493 (7.4)	
Yes	66,029 (92.3)	675 (97.1)	3318 (94.6)	8260 (93.9)	35,067 (91.5)	18,709 (92.6)	
Salt intake, N (%)							<0.001
Low	6377 (8.9)	50 (7.2)	334 (9.5)	808 (9.2)	3453 (9.0)	1732 (8.6)	
Medium	57,717 (80.7)	602 (86.6)	2893 (82.5)	7171 (81.5)	30,760 (80.3)	16,291 (80.6)	
High	7432 (10.4)	43 (6.2)	281 (8.0)	822 (9.3)	4107 (10.7)	2179 (10.8)	
Anti-hypertensives, N (%)	7186 (10.0)	39 (5.6)	354 (10.1)	1000 (11.4)	3962 (10.3)	1831 (9.1)	<0.001
Hypertension, N (%)	30,185 (42.2)	251 (36.1)	1500 (42.8)	3838 (43.6)	16,299 (42.5)	8297 (41.1)	<0.001
BMI, mean ± SD, kg/m^2^	25.0 ± 3.4	24.2 ± 3.4	24.3 ± 3.4	24.6 ± 3.4	25.0 ± 3.4	25.2 ± 3.5	<0.001
eGFR, median (IQR), mL/min/1.73 m^2^	80.7 (68.3–94.5)	82.1 (68.4–97.6)	80.3 (67.0–94.9)	81.2 (68.3–94.3)	82.5 (69.3–96.3)	77.7 (66.7–90.4)	<0.001
Creatinine, mean ± SD, μmol/L	90.7 ± 20.0	86.3 ± 19.8	88.2 ± 20.9	88.3 ± 21.9	89.1 ± 19.3	95.3 ± 19.5	<0.001
SUA, median (IQR), μmol/L	285.0 (234.0–342.0)	286.0 (240.0–334.0)	289.0 (236.0–347.0)	288.0 (233.0–345.0)	283.9 (233.0–341.0)	286.0 (235.0–343.0)	<0.001
BUN, mean ± SD, mmol/L	5.7 ± 1.5	5.4 ± 1.5	5.4 ± 1.5	5.4 ± 1.6	5.7 ± 1.5	5.9 ± 1.4	<0.001
TC, mean ± SD, mmol/L	5.0 ± 1.0	5.0 ± 1.0	5.0 ± 1.0	5.0 ± 1.0	5.0 ± 1.0	5.0 ± 1.0	<0.001
TG, median (IQR), mmol/L	1.2 (0.9–1.8)	1.3 (0.9–1.9)	1.2 (0.9–1.8)	1.2 (0.9–1.8)	1.2 (0.9–1.8)	1.2 (0.9–1.8)	0.61
HDL-C, mean ± SD, mmol/L	1.6 ± 0.4	1.6 ± 0.4	1.6 ± 0.4	1.6 ± 0.4	1.5 ± 0.4	1.6 ± 0.4	<0.001
LDL-C, mean ± SD, mmol/L	2.3 ± 0.9	2.3 ± 0.8	2.3 ± 0.8	2.2 ± 0.9	2.3 ± 0.9	2.3 ± 0.9	<0.001
hs-CRP, median (IQR), mg/L	0.8 (0.3–2.2)	0.7 (0.2–1.6)	0.9 (0.3–2.3)	0.8 (0.3–2.3)	0.8 (0.3–2.1)	0.8 (0.3–2.4)	<0.001
Hematocrit (%), mean ± SD, L/L	44.1 ± 4.8	43.4 ± 5.0	43.1 ± 4.8	43.0 ± 4.7	43.8 ± 4.6	45.2 ± 5.0	<0.001
FBG, mean ± SD, mmol/L	5.1 ± 0.7	5.0 ± 0.7	5.0 ± 0.7	5.0 ± 0.7	5.1 ± 0.7	5.0 ± 0.7	<0.001
SBP, mean ± SD, mmHg	130.3 ± 20.8	126.8 ± 19.0	129.9 ± 20.9	131.0 ± 21.3	130.6 ± 20.7	129.6 ± 20.8	<0.001
DBP, mean ± SD, mmHg	83.2 ± 11.7	81.0 ± 10.8	82.1 ± 11.6	82.9 ± 11.6	83.4 ± 11.7	83.3 ± 11.8	<0.001

**Table 2:** Associations of baseline and time-varying hydration status with incident type 2 diabetes using multivariable and time-dependent Cox regression models among the study participants during the follow-up period (2006–2020).

	Group 1	Group 2	Group 3	Group 4	Group 5	*p* for Trend
**Baseline**						
Individuals	695	3508	8801	38320	20202	
Cases, n (%)	78 (11.22)	451 (12.86)	1174 (13.34)	5951 (15.53)	3430 (16.98)	
Incidence rate, per 1000 person years	9.13	10.59	10.96	12.71	13.85	
Model 1	Reference	1.16 (0.91–1.48)	1.20 (0.95–1.52)	1.39 (1.11–1.74)	1.57 (1.21–1.90)	<0.001
Model 2	Reference	1.18 (0.93–1.50)	1.20 (0.95–1.50)	1.34 (1.07–1.67)	1.37 (1.10–1.72)	<0.001
Model 3	Reference	1.16 (0.91–1.48)	1.17 (0.92–1.47)	1.30 (1.04–1.63)	1.38 (1.10–1.74)	<0.001
**Time-varying**						
Model 1	Reference	1.09 (0.99–1.21)	1.19 (1.08–1.31)	1.32 (1.20–1.45)	1.41 (1.28–1.55)	<0.001
Model 2	Reference	1.08 (0.97–1.20)	1.15 (1.04–1.27)	1.26 (1.14–1.38)	1.36 (1.24–1.50)	<0.001
Model 3	Reference	1.09 (0.98–1.21)	1.16 (1.05–1.28)	1.26 (1.14–1.38)	1.33 (1.21–1.47)	<0.001

Note: Group 1: 1.000 ≤ USG < 1.010; Group 2: 1.010 ≤ USG < 1.015; Group 3: 1.015 ≤ USG < 1.020; Group 4: 1.020 ≤ USG < 1.030; Group 5: USG ≥ 1.030. Model 1: crude model; Model 2: adjusted for age, gender, BMI, education, smoking, drinking status, physical activity, and intake of salt based on model 1; Model 3: further adjusted for history of hypertension, total cholesterol (TC), triglyceride (TG), C-reactive protein (CRP), serum uric acid (SUA), eGFR, blood urea nitrogen (BUN), plasma creatinine (Cre), and hematocrit based on model 2; time-varying covariate adjustment for covariates except for gender and education in time-dependent Cox regression models.

## Data Availability

The Kailuan cohort study database is not publicly available and will be available from the corresponding author S.W. on reasonable request.

## Supplementary Materials

The following supporting information can be downloaded at https://www.mdpi.com/article/10.3390/nu16111643/s1, Figure S1: Flow chart of the study population; Figure S2: Venn diagram displaying the overlapping combinations of fasting blood glucose, self-report of a physician diagnosis, or self-reported uses of antidiabetic medication in the study participants who developed type 2 diabetes over the 15 years; Table S1: Full results of associations between baseline USG and risk of T2DM in the full-adjusted Cox regression model; Table S2: Full results of associations between time-varying USG and risk of T2DM in the full-adjusted Cox regression model; Table S3: Subgroup analyses of the associations between baseline USG levels and type 2 diabetes incidence among adults from the Kailuan Cohort study during the follow-up period (2006–2020); Table S4: Sensitivity analysis of the association between baseline USG levels and type 2 diabetes risks during the follow-up period (2006–2020) after excluding those with events at first follow-up and severe malnutrition at baseline; Table S5: Sensitivity analysis of the association between different hydration status and type 2 diabetes risks during the follow-up period (2006–2020) with an additional cutoff for severe dehydration; Table S6: Sensitivity analysis of associations between different hydration status and incident type 2 diabetes among the total population directly classified into four hydration status subgroups during the follow-up period (2006–2020); Table S7: Sensitivity analysis of the associations between severe dehydration and incident type 2 diabetes compared with participants with non-severe dehydration in clinical practice.
