# Supplementary material for: Association between Urine Specific Gravity as a Measure of Hydration Status and Risk of Type 2 Diabetes: The Kailuan Prospective Cohort Study
Source: Nutrients. 2024 May 27;16(11):1643. doi: 10.3390/nu16111643 (PMC11174895; doi:10.3390/nu16111643)
Supplement: Supplementary file 1 [file nutrients-16-01643-s001.zip › nutrients-2981729-supplementary.pdf]

Supplementary Materials

Figure S1: Flow chart of the study population.

Figure S2: Venn diagram displaying the overlapping combinations of fasting blood glucose, self-report of a physician diagnosis, or self-reported uses of antidiabetic medication in the study participants who developed type 2 diabetes over the 15 years.

Table S1: Full results of associations between baseline USG and risk of T2DM in the full-adjusted Cox regression model.

Table S2: Full results of associations between time-varying USG and risk of T2DM in the full-adjusted Cox regression model.

Table S3: Subgroup analyses of the associations between baseline USG levels and type 2 diabetes incidence among adults from the Kailuan Cohort study during the follow-up period (2006-2020).

Table S4: Sensitivity analysis of the association between baseline USG levels and type 2 diabetes risks during the follow-up period (2006-2020) after excluding those with events at first follow-up and severe malnutrition at baseline.

Table S5: Sensitivity analysis of the association between different hydration status and type 2 diabetes risks during the follow-up period (2006-2020) with an additional cutoff for severe dehydration.

Table S6: Sensitivity analysis of the associations between different hydration status and incident type 2 diabetes among the total population directly classified into four hydration status subgroups during the follow-up period (2006-2020).

Table S7: Sensitivity analysis of the associations between severe dehydration and incident type 2 diabetes compared with participants with non-severe dehydration in clinical practice.

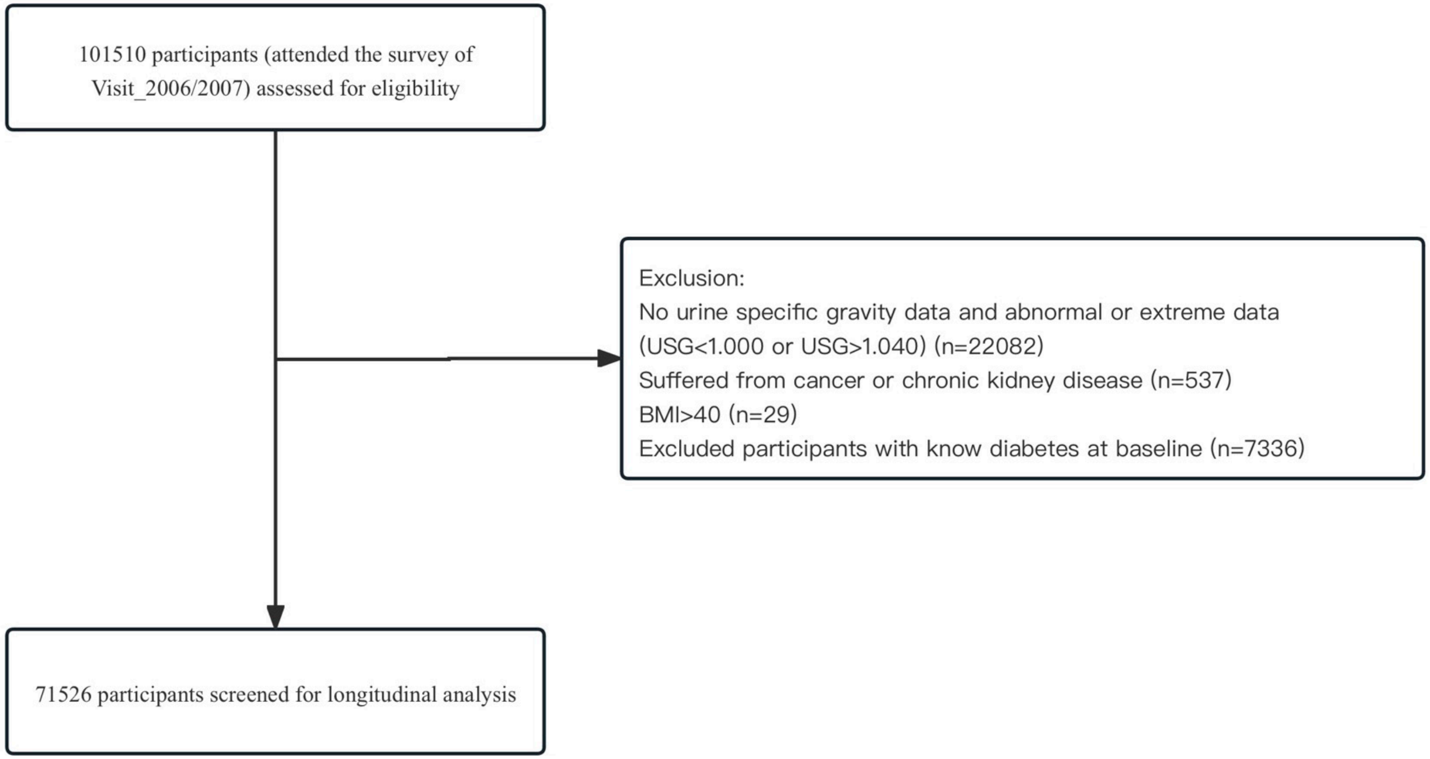

Figure S1: **Flow chart of the study population.**

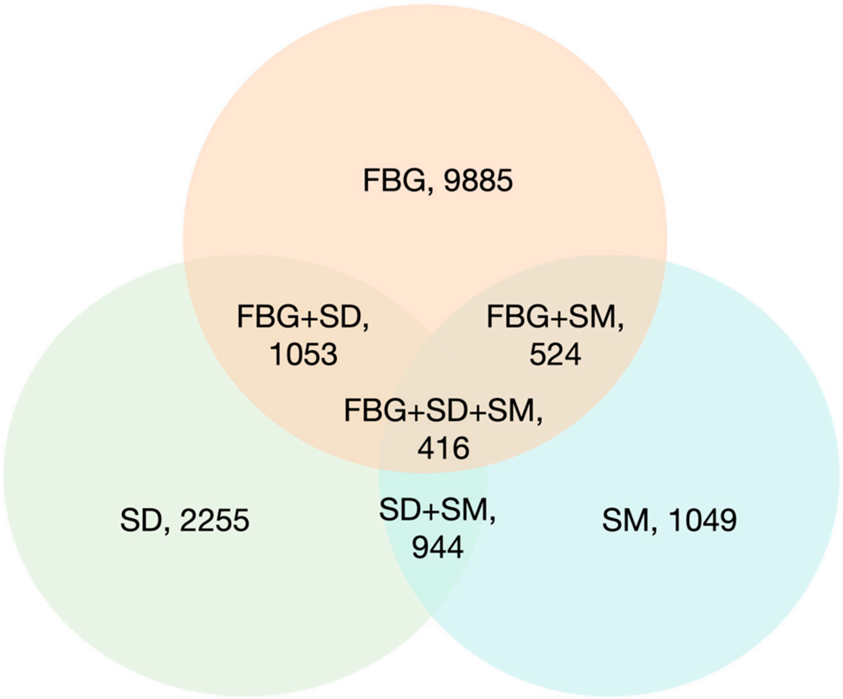

Figure S2: **Venn diagram displaying the overlapping combinations of fasting blood glucose, self-report of a physician diagnosis, or self-reported uses of anti-diabetic medication in the study participants who developed type 2 diabetes over 15 years (N = 11084)**

Note: FBG, fasting blood glucose; SD, self-report of a physician diagnosis; SM, self-reported uses of antidiabetic medication

Table S1. Full results of associations between baseline USG and risk of T2DM in the full-adjusted Cox regression model.

Variable	HR (95% CI)	*P* value
USG group 2	1.16 (0.91-1.48)	0.280
USG group 3	1.17 (0.92-1.47)	0.248
USG group 4	1.30 (1.04-1.63)	0.035
USG group 5	1.38 (1.10-1.74)	0.005
Age (≥65 years)	1.12 (1.07-1.17)	<0.001
Gender (Male)	1.40 (1.29-1.52)	<0.001
Education (High school and above)	0.86 (0.81-0.90)	<0.001
Overweight (24≤BMI<28)	1.62 (1.55-1.70)	<0.001
Obesity (≥28)	2.38 (2.25-2.51)	<0.001
Smoking (yes)	1.05 (1.00-1.09)	0.058
Drinking (yes)	1.01 (0.95-1.06)	0.945
Physical activity (yes)	0.91 (0.84-0.97)	0.005
Salt intake (6-10g/day)	1.04 (0.97-1.11)	0.326
Salt intake (>10g/day)	1.09 (1.01-1.20)	0.048
Triglyceride	1.57 (1.52-1.62)	<0.001
Total cholesterol	1.06 (1.04-1.08)	<0.001
C-reactive protein	1.08 (1.07-1.10)	<0.001
Serum Uric Acid	1.00 (1.00-1.00)	0.790
eGFR	0.98 (0.98-0.99)	<0.001
Blood urea nitrogen	0.99 (0.98-1.00)	0.125
Plasma creatinine	0.99 (0.98-0.99)	<0.001
Hematocrit	2.08 (1.32-3.28)	0.002
History of hypertension	2.17 (2.04-2.31)	<0.001

Table S2. Full results of associations between time-varying USG and risk of T2DM in the full-adjusted Cox regression model.

Variable	HR (95% CI)	*P* value
USG group 2	1.09 (0.98-1.21)	0.280
USG group 3	1.16 (1.05-1.28)	0.248
USG group 4	1.26 (1.14-1.38)	0.035
USG group 5	1.33 (1.21-1.47)	0.005
Age (≥65 years)	1.04 (1.02-1.07)	<0.001
Gender (Male)	1.16 (1.12-1.19)	<0.001
Education (High school and above)	0.91 (0.90-0.92)	<0.001
Overweight (24≤BMI<28)	1.42 (1.38-1.45)	<0.001
Obesity (≥28)	1.90 (1.85-1.95)	<0.001
Smoking	1.09 (1.07-1.11)	<0.001
Drinking	1.01 (0.98-1.03)	0.695
Physical activity (no)	1.02 (1.01-1.05)	0.034
Salt intake (6-10g/day)	0.98 (0.95-1.01)	0.172
Salt intake (>10g/day)	0.99 (0.95-1.03)	0.725
Triglyceride	1.04 (1.04-1.04)	<0.001
Total cholesterol	1.03 (1.03-1.03)	<0.001
C-reactive protein	1.01 (1.01-1.01)	<0.001
Serum Uric Acid	1.00 (1.00-1.00)	0.072
eGFR	0.93 (0.89-0.98)	0.003
Blood urea nitrogen	1.00 (1.00-1.00)	0.846
Plasma creatinine	1.00 (1.00-1.00)	<0.001
Hematocrit	1.00 (1.00-1.00)	<0.001
History of hypertension	1.54 (1.51-1.57)	<0.001

Supplementary Table S3: Subgroup analyses of the associations between baseline USG levels and type 2 diabetes incidence among adults from the Kailuan Cohort study during the follow-up period (2006-2020).

	N	Group 1	Group 2	Group 3	Group 4	Group 5	*P* for trend	*P* for interaction
		HR (95%CI)	HR (95%CI)	HR (95%CI)	HR (95%CI)	HR (95%CI)		
**Sex**								<.001
**Female**	25632	1(ref)	1.13 (0.73, 1.75)	1.06 (0.70, 1.61)	1.31 (1.01, 1.78)	1.39 (1.03, 1.93)	<.001	
**Male**	25428	1(ref)	1.20 (0.89, 1.61)	1.24 (0.94, 1.64)	1.37 (1.04, 1.80)	1.46 (1.11, 1.92)	<.001	
**Age**								<.001
**<65**	35508	1(ref)	1.11 (0.86, 1.43)	1.13 (0.89, 1.43)	1.25 (1.01, 1.60)	1.36 (1.07, 1.71)	<.001	
**≥65**	15552	1(ref)	2.21 (0.81, 6.05)	2.11 (0.89, 5.69)	2.50 (1.02, 6.70)	2.78 (1.03, 7.49)	<.001	
**Smoking status**								0.21
**Non-smoker**	26271	1(ref)	1.02 (0.78, 1.34)	1.05 (0.81, 1.35)	1.18 (0.92, 1.51)	1.33 (1.04, 1.71)	<.001	
**Smoker**	22013	1(ref)	2.02 (1.09, 3.74)	1.95 (1.07, 3.56)	2.17 (1.20, 3.94)	2.21 (1.22, 4.01)	<.001	
**Drinking status**								0.18
**No**	14138	1(ref)	1.06 (0.80, 1.39)	1.08 (0.83, 1.40)	1.21 (0.94, 1.56)	1.35 (1.05,1.74)	<.001	
**Yes**	29646	1(ref)	1.60 (0.93, 2.78)	1.56 (0.92, 2.67)	1.74 (1.03, 2.95)	1.83 (1.08, 3.09)	<.001	

NOTE: The subgroup analyses were analyzed by the multivariable Cox regression model and adjusted for all potential variables, including baseline age, gender, BMI (categorical), education, smoking, drink, physical activity, intake of salt, history of hypertension, total cholesterol (TC), triglyceride (TG), C-reactive protein (CRP), serum uric acid (SUA), estimated glomerular filtration rate (eGFR), blood urea nitrogen (BUN), plasma creatinine (Cre) and hematocrit. The hydration status was measured by urine specific gravity concentration and categorized into five groups: Group 1: 1.000≤USG<1.010; Group 2: 1.010≤USG<1.015; Group 3: 1.015≤USG<1.020; Group 4: 1.020≤USG<1.030; Group 5: USG≧1.030. The test for trend was based on the variable containing the median value for each quartile. The interaction effect was evaluated by the likelihood ratio test.

Supplementary Table S4: Sensitivity analysis of associations between baseline USG levels and type 2 diabetes risks during the follow-up period (2006-2020) after excluding those with events at first follow-up and severe malnutrition at baseline.

	Categories of urine specific gravity, HRs (95% CIs)					*P* for trend
	Group 1	Group 2	Group 3	Group 4	Group 5	
**Excluded participants with events in the first follow-up visit (N=2565)**						
Case, n (%)	60 (8.86)	341 (10.64)	285 (10.40)	4541 (12.30)	2692 (13.83)	
Incidence rate, per 1000 person-years	7.06	8.06	8.31	9.76	10.94	
Model 1	Reference	1.14 (0.87,1.50)	1.18 (0.91,1.53)	1.38 (1.07,1.78)	1.52 (1.21,1.90)	<.001
Model 2	Reference	1.18 (0.89,1.55)	1.19 (0.91,1.54)	1.33 (1.03,1.72)	1.37 (1.10,1.72)	<.001
Model 3	Reference	1.17 (0.88,1.54)	1.17 (0.90,1.53)	1.31 (1.02,1.70)	1.38 (1.10,1.74)	<.001
**Excluded participants with BMI less than 18 (severely malnourished) at baseline (N=123)**						
Case, n (%)	78 (11.26)	451 (12.89)	1173 (13.36)	5951 (15.56)	3429 (16.99)	
Incidence rate, per 1000 person-years	9.15	10.62	10.97	12.73	13.98	
Model 1	Reference	1.16 (0.91,1.48)	1.20 (0.95,1.51)	1.39 (1.11,1.74)	1.50 (1.20,1.88)	<.001
Model 2	Reference	1.16 (0.91,1.47)	1.14 (0.91,1.44)	1.26 (1.02,1.58)	1.38 (1.10,1.73)	<.001
Model 3	Reference	1.14 (0.89,1.46)	1.14 (0.90,1.43)	1.26 (1.01,1.58)	1.36 (1.09,1.58)	<.001

Note: The sensitivity analyses were analyzed by the multivariable Cox regression model. Group 1: 1.000 ≤USG<1.010 g/mL; Group 2: 1.010≤USG<1.015 g/mL; Group 3: 1.015≤USG<1.020 g/mL; Group 4: 1.020≤USG<1.030 g/mL; Group 5: USG≧1.030 g/mL.

Model 1: crude model; Model 2: adjusted for age, gender, BMI (categorical), education, smoke, drink, physical activity and intake of salt based on model 1;

Model 3: further adjusted for history of hypertension, total cholesterol (TC), triglyceride (TG), C-reactive protein (CRP), Serum Uric Acid (SUA), estimated glomerular filtration rate (eGFR), Blood urea nitrogen (BUN), Plasma creatinine (Cre) and hematocrit based on model 2.

Supplementary Table S5: Sensitivity analysis of the association between different hydration status and type 2 diabetes risks during the follow-up period (2006-2020) with an additional cutoff for severe dehydration.

	Categories of urine specific gravity, HRs (95% CIs)					*P* for trend
	**Group 1**	**Group 2**	**Group 3**	**Group 4**	**Group 5**	
Case, n (%)	78 (11.22)	451 (12.86)	1174 (13.34)	2494 (14.74)	6887 (16.56)	
Incidence rate, per 1000 person-years	9.13	10.59	10.96	12.04	13.54	
Model 1	Reference	1.16 (0.91,1.48)	1.20 (0.95,1.51)	1.32 (1.05,1.65)	1.48 (1.19,1.85)	<.001
Model 2	Reference	1.18 (0.93,1.50)	1.20 (0.95,1.50)	1.28 (1.03,1.61)	1.35 (1.08,1.69)	<.001
Model 3	Reference	1.15 (0.90,1.47)	1.16 (0.92,1.47)	1.25 (1.01,1.59)	1.34 (1.07,1.68)	<.001

Note: This sensitivity analysis adjusted the threshold of the severe dehydration group (Group 5) from 1.030 to 1.025 and reanalyzed the relationship between baseline USG levels and T2D risk based on multivariable Cox regression model.

Group 1: 1.000 ≤USG≤1.010 g/mL; Group 2: 1.010≤USG<1.015 g/mL; Group 3: 1.015≦USG<1.020 g/mL; Group 4: 1.020≦USG<1.025 g/mL; Group 5: USG≧1.025 g/mL.

Model 1: crude model;

Model 2: adjusted for age, gender, BMI (categorical), education, smoking, drink, physical activity, and intake of salt based on model 1;

Model 3: further adjusted for history of hypertension, total cholesterol (TC), triglyceride (TG), C-reactive protein (CRP), Serum Uric Acid (SUA), estimated glomerular filtration rate (eGFR), Blood urea nitrogen (BUN), Plasma creatinine (Cre) and hematocrit based on model 2.

Supplementary Table S6: Sensitivity analysis of associations between different hydration status and incident type 2 diabetes among the total population directly classified into four hydration status subgroups during the follow-up period (2006-2020).

	Group 1	Group 2	Group 3	Group 4	P for trend
Individuals	4194	8803	16913	41616	
Cases, n (%)	527 (12.57)	1176 (13.36)	2494 (14.75)	6887 (16.55)	
Incidence rate, per 1000 person-years	10.33	10.97	12.05	13.53	
Model 1	Reference	1.06 (0.96-1.18)	1.17 (1.06-1.28)	1.31 (1.20-1.43)	**<.001**
Model 2	Reference	1.02 (0.92-1.13)	1.09 (0.99-1.20)	1.18 (1.08-1.29)	**<.001**
Model 3	Reference	1.02 (0.92-1.14)	1.10 (1.00-1.21)	1.20 (1.09-1.31)	**<.001**

Note: The sensitivity analysis combined the dehydrated (1.020≤USG<1.030 g/mL) and severely dehydrated (USG≧1.030 g/mL) groups and reanalyzes the relationship between baseline USG levels and T2D risk based on multivariable Cox regression model.

Group 1: 1.000≤USG<1.010; Group 2: 1.010≤USG≤1.015; Group 3: 1.015≤USG≤1.020; Group 4: USG≧1.020.

Model 1: crude model;

Model 2: adjusted for age, gender, BMI (categorical), education, smoking, drinking status, physical activity, and intake of salt based on model 1;

Model 3: further adjusted for history of hypertension, total cholesterol (TC), triglyceride (TG), C-reactive protein (CRP), Serum Uric Acid (SUA), eGFR, Blood urea nitrogen (BUN), Plasma creatinine (Cre) and hematocrit based on model 2.

Supplementary Table S7: Sensitivity analysis of the associations between severe dehydration and incident type 2 diabetes compared with participants with non-severe dehydration in clinical practice.

**Hydration index**	Individuals	Events, n (%)	Incidence rate	Model 1		Model 2		Model 3	
			per 1000-person years	HR (95% CI)	*P* value	HR (95% CI)	*P* value	HR (95% CI)	*P* value
**Urine specific gravity (g/mL)**									
USG<1.030	51324	7654 (14.91)	12.22	Reference		Reference		Reference	
USG≧1.030	20202	3430 (16.97)	13.85	1.13 (1.09-1.18)	**<.001**	1.10 (1.06-1.15)	**<.001**	1.11 (1.07-1.16)	**<.001**
